# Power management technologies for triboelectric nanogenerators

**DOI:** 10.1557/s43577-025-00860-8

**Published:** 2025-02-20

**Authors:** Sijun Du, Philippe Basset, Hengyu Guo, Dimitri Galayko, Armine Karami

**Affiliations:** 1 Department of Microelectronics, Delft University of Technology, Delft, The Netherlands; 2 Centre National de la Recherche Scientifique ESYCOM Lab, Université Gustave Eiffel/ESIEE Paris, Marne-la-Vallée, France; 3 College of Physics, Chongqing University, Chongqing, China; 4 Laboratory LIP6, Sorbonne Université, Paris, France; 5 ESYCOM Laboratory, Centre National de la Recherche Scientifique, Paris, France

**Keywords:** Triboelectric nanogenerator (TENG), Energy harvesting, Power conversion, Mechanical energy, Power management, Wearable electronics

## Abstract

A triboelectric nanogenerator (TENG) is a novel device that utilizes contact electrification and electrostatic induction to convert mechanical energy into electrical energy. Its characteristics include high energy density and flexibility, enabling self-powering of electronic devices by harvesting mechanical energy from the environment. Its applications include biomedical devices, wearable electronics, and Internet-of-Things (IoT) sensors. Despite these advantages, extracting electrical energy from TENG remains challenging due to its time-varying nature and low internal capacitance. Effective power-management techniques are essential for TENG energy-harvesting systems, yet research on dedicated integrated power-conversion methods is currently limited. Given the growing interest in TENG, a comprehensive exploration of energy-harvesting systems is critically necessary. This article synthesizes and compares current advancements in triboelectric energy-harvesting systems, emphasizing strategies to enhance output power through various power-conversion techniques. Additionally, it explores techniques employed in other energy-harvesting systems to inspire innovative approaches in TENG system design.

**Graphical abstract:**

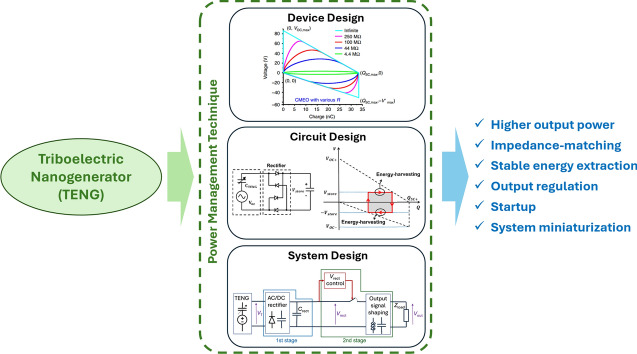

## Introduction

In today’s world, electronic devices, including those in the Internet-of-Things (IoT) and biomedical sectors, have become essential for enhancing convenience and quality of life. These devices typically rely on batteries, which eventually need to be replaced or recharged. This has led to significant interest in battery-free systems over the past decade, focusing on energy-harvesting technologies, capturing and converting ambient energy into usable electricity.^1^ Kinetic energy is particularly abundant, and interest in harnessing it has grown substantially in recent decades.^2^ Piezoelectric energy harvesting, a widely used method, converts mechanical movements and vibrations into electrical energy by relying on the oscillations of internal cantilevers or plates.^3^ However, this method is most effective with regular and stable mechanical vibrations, and it performs poorly with irregular and unpredictable movements such as human motion and tidal forces. To overcome these limitations and enable kinetic energy harvesting in a broader range of scenarios, the triboelectric nanogenerator (TENG) was introduced.^4^

TENGs operate on the principles of triboelectrification (contact electrification) and electrostatic induction.^5^ Triboelectrification occurs when two different materials come into contact and exchange electrical charges. Changes in the distance or contact area between these materials generate an electric potential due to the displacement current caused by the movement of static charges. This mechanism shows promise for sensing displacement and converting mechanical energy into electrical energy. However, despite its potential, there are several challenges that need to be addressed, including efficient energy conversion, storage, and delivery. These challenges are critical to ensuring that TENGs can reliably and effectively support real-world applications.

Since its introduction in 2012,^4^ TENG technology has attracted increasing attention for its potential in energy harvesting. TENGs offer high flexibility and energy density, making them more suitable for miniaturization and planar integration compared to piezoelectric and electrostatic harvesters. Furthermore, TENGs are not limited by resonant frequency, allowing them to generate more power from low and irregular vibrations.^6^ TENGs have been successfully used to harvest mechanical energy from human body movements when integrated into textiles, bracelets, and even internal organs, providing a reliable power source for low-power wearable and implantable devices, such as IoT environmental monitors, health monitors, and human–machine interfaces.^7–9^ Additionally, TENGs have been explored for harvesting energy from natural sources such as tides, waves, and wind.

To efficiently extract energy from the previously mentioned application scenarios, improvements in the power-management system play a critical role in enhancing the overall performance of TENG-based energy harvesters. Unlike material innovations, which focus on enhancing triboelectric properties and durability, power-management systems are vital for maximizing energy extraction, storage, and delivery efficiency, particularly from irregular and low-frequency vibrations. Efficient and adaptive power-management strategies help optimize the harvested energy by addressing issues such as impedance matching, energy storage, and real-time power conditioning.

This article will review recent innovations in power-management systems for TENGs, covering device designs, energy extraction circuits, and system-level optimizations. These aspects are crucial for enabling practical applications of TENG technology in both wearable and environmental settings, ensuring that the energy generated can be efficiently converted and utilized. By providing a comprehensive overview of the current progress and identifying future directions, this article emphasizes the critical role of power management in bridging the gap between laboratory research and real-world implementation, further highlighting the importance of TENGs in advancing sustainable energy solutions.

## Device design and principle for tuning the nanogenerator output characteristic

TENGs operate on the fundamental principles of contact electrification and electrostatic induction. Contact electrification occurs when two different materials come into contact and exchange electrical charges due to differences in their electron affinities. When these materials are subsequently separated, the charge imbalance creates an electric potential difference. Electrostatic induction comes into play when an external load is connected, enabling the flow of induced charges in the circuit. Together, these mechanisms convert mechanical energy into electrical energy, forming the basis of TENG operation. These principles dictate the output characteristics of TENGs, including their voltage, charge, and energy-generation behaviors.

The high impendence of TENGs, which ranges from megaohms to gigaohms, severely limits their energy utilization efficiency when directly powering electronic devices. Therefore, tuning the output characteristic is essential for maximizing the energy extraction of TENGs. Basically, the energy output (*E*) of a TENG can be expressed through the relationship between the accumulated voltage (*V*) and the transferred charge (*Q*):^10^

1 $$E = {\oint }V \cdot dQ.$$

**Figure 1:**
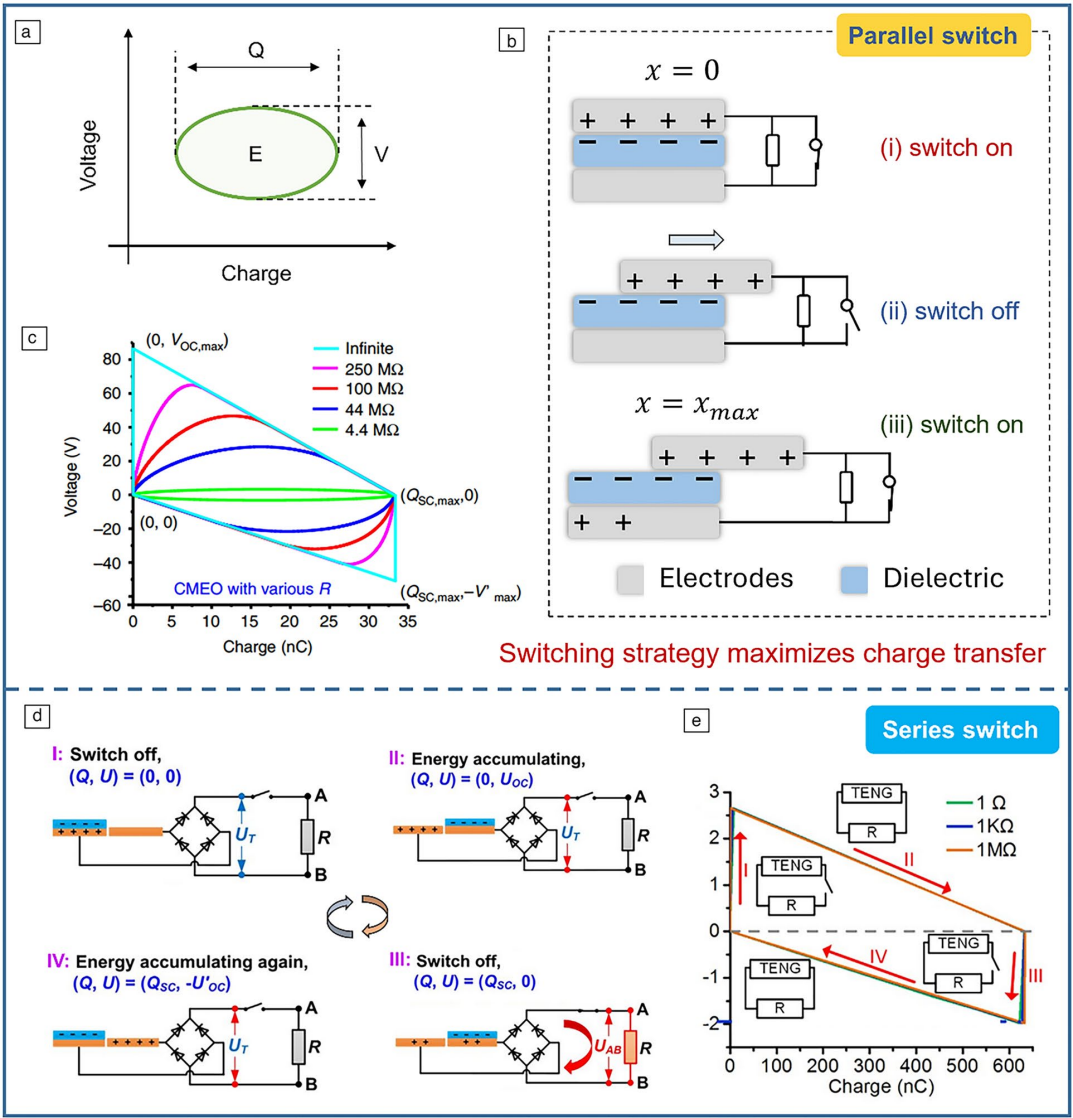
Maximizing energy extraction from triboelectric nanogenerators (TENGs). (a) *VQ* curve of a TENG under a certain external load. (b) Changes in switch status with TENG movement. (c) The optimized *VQ* curves with various load resistances.^7^ (d) Operating process in one cycle for releasing maximum energy from TENG and transferring it to a resistor by sequential switching.^11^ (e) The *U*–*Q* plot of the TENG in the maximized energy transfer cycle.^12^ CMEO, cycles for maximized energy output.

The steady-state output of TENGs in response to mechanical triggering is periodic, forming a closed loop in the *V*–*Q* plot. According to Equation 1, the loop area represents the energy output per cycle (**Figure** 1a). Theoretically, as the external load increases, the cycling voltage rises, but the cycling charge decreases. Even if an optimal load is matched to achieve maximum energy output, this maximum output is still unfulfilled due to insufficient charge transfer.

To solve this, Zi et al. proposed a parallel switch design,^7^ as illustrated in Figure 1b. Initially, the switch is off during the top electrode moves from $x=0$ to $x={x}_{max}$, and closes at maximum displacement. As the electrode returns from ${x}_{max}$ to $x=0$, the switch resets to the off state and turns to the on state when the electrode reaches the starting position. The double-switching process during the TENG working cycle completes the sufficient charge transfer under any external resistance. Figure 1c demonstrates the effectiveness of this strategy, and the ultimate energy output of TENG can be achieved under the infinite resistance (trapezoid curve in Figure 1c).

Accordingly, in 2017, Xi et al. introduced a serial switch design (Figure 1d).^11^ Consistent with the triggering conditions of the parallel switch (Figure 1b), whenever the slider displacement reaches its maximum distance, that is, when the voltage accumulation reaches its peak, the switch closes. The key difference here is that the switch is in series after the rectifier, enabling the entire energy of the TENG to be transferred to the external load. Cheng et al. further extended this energy maximization mechanism to the contact-separation mode TENG, verifying the broad applicability of this strategy, and demonstrated that the output energy is maximized regardless of the value of the connected load resistance (Figure 1e).^12^

**Figure 2:**
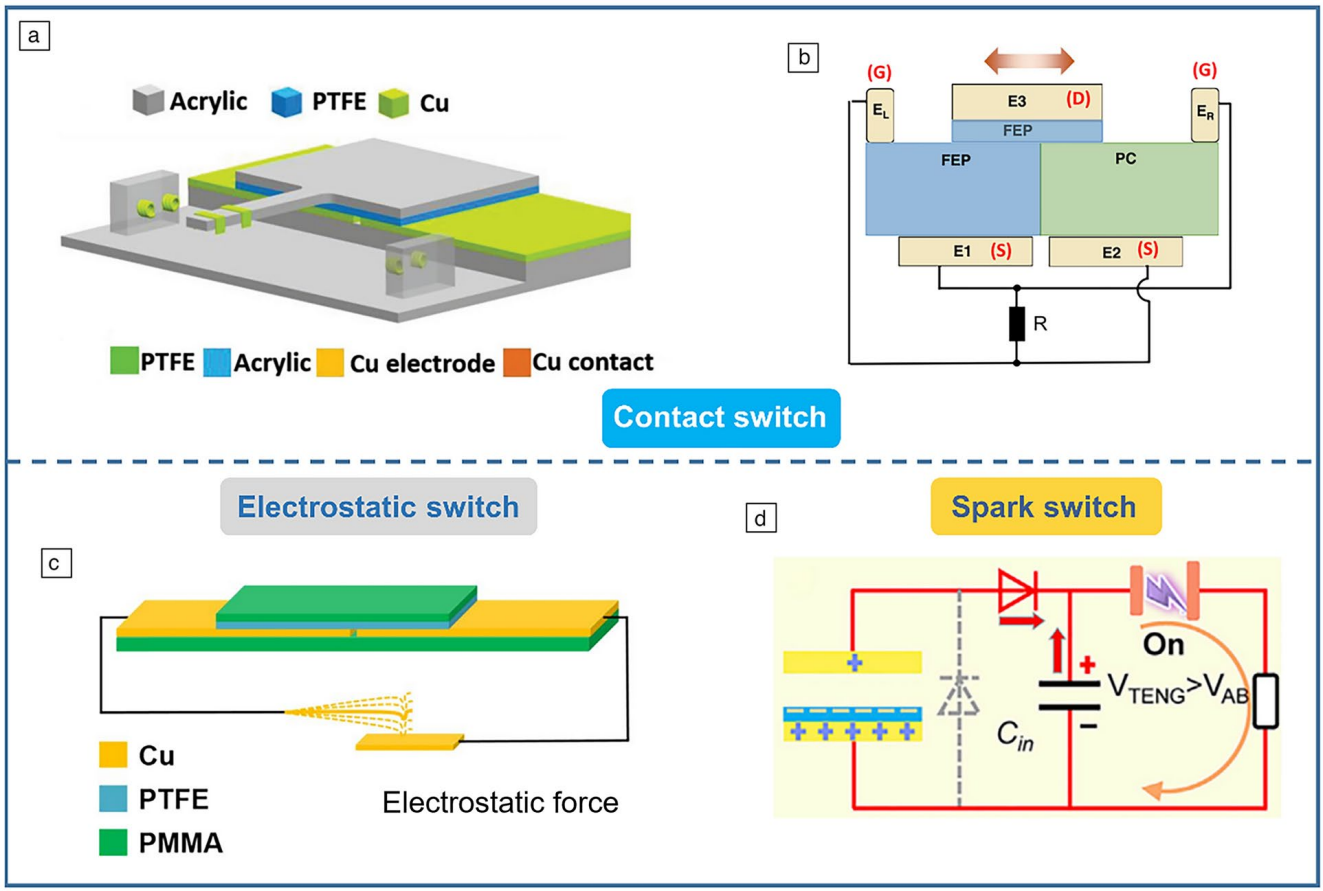
Different switch designs. (a) Unidirectional switch.^13^ (b) Transistor-like triboelectric nanogenerator (TENG) with reverse charge enhancement.^14^ (c) Electrostatic vibration switch.^15^ (d)  Plate-to-plate spark discharge switch.^16^

Based on this principle, researchers have developed various switches to accomplish the previously discussed energy process. Qin et al. created a TENG with a unidirectional switch (**Figure** 2a) that closes when the slider reaches maximum displacement, releasing maximum energy.^13^ Wu et al. further enhanced sliding mode TENGs with a transistor-like design and reverse charge enhancement (Figure 2b), achieving high instantaneous power density.^14^ Yang et al. designed an electrostatic vibration switch that uses electrostatic force for switching, improving TENG peak output by 4700 times (Figure 2c).^15^ However, mechanical switches face wear and tear. To address this issue, Wang et al. proposed a plate-to-plate spark discharge switch (Figure 2d).^16^ This switch charges a prestage capacitor C_in_ during TENG separation. When the voltage across C_in_ exceeds a threshold, it breaks down the air gap, forming a conductive channel that releases energy. By adjusting the gap distance, optimal output energy is achieved.

These advancements in switch design mark the first transformation of the TENGs' output form, shifting from the original output to high-frequency pulse signals that are easier for backend circuits to manage. Besides, specific device design, a process referred to as the preself-management, also contributes to tuning the output characteristics of TENGs. Typically, minimizing the output electrode spacing and subdividing more electrode units can lower the internal resistance of TENG devices,^17^ thereby better matching the back-end power management process.

## Power-management circuits for TENGs

### Passive rectifiers

**Figure 3:**
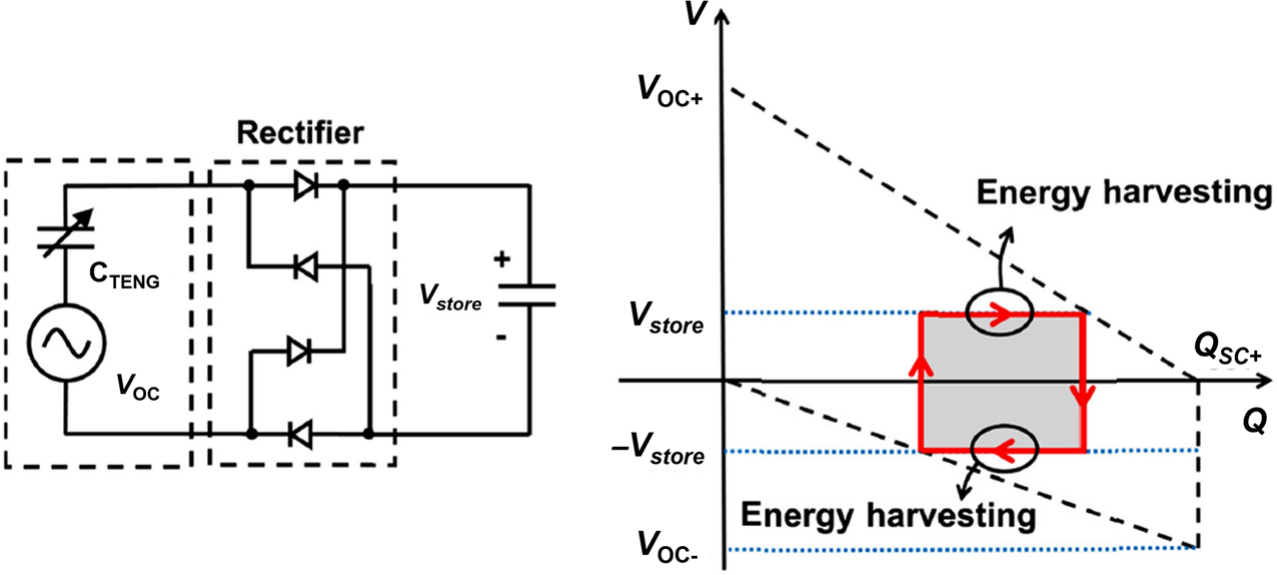
The configuration and *V*–*Q* plot of the standard energy-harvesting circuit in triboelectric energy harvesting. TENG, triboelectric nanogenerator.

The output from a TENG is AC energy, which requires rectifiers to convert it to DC energy suitable for powering electronic devices. Passive rectifiers are frequently used because they are simple, stable, and capable of easy cold-startup, although they often have lower energy-conversion efficiencies. These rectifiers are built using passive elements such as capacitors and diodes or transistors acting as diodes. The two primary types of rectifier configurations are the half bridge and the full bridge. The half bridge rectifier includes two diodes; one diode allows current to flow in one direction, while the other grounds the AC input. Conversely, the full bridge rectifier (FBR) uses two half bridge rectifiers to manage current flow in both directions, thus allowing for more efficient energy extraction in a single cycle, which is why it is more commonly used in energy harvesting. The energy-harvesting interface that consists of an FBR and a rectification capacitor is referred to as a standard energy-harvesting circuit, as shown in **Figure** 3.^18^ In practical applications, deploying an FBR in triboelectric energy harvesting presents distinct challenges depending on the application scenario. In low-power applications, where input voltages are low, it is crucial for the rectifier to minimize leakage current and conduction losses. In high-power applications, the rectifier must be capable of handling high voltages, although the voltage drop across the diode is relatively insignificant.

**Figure 4:**
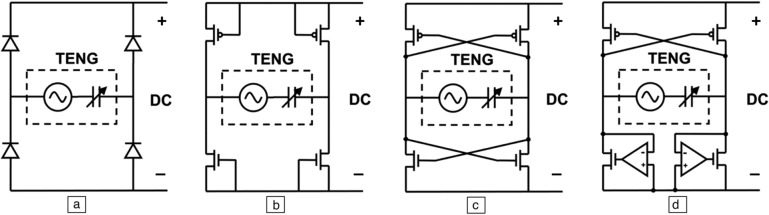
Different configurations of full bridge rectifiers in triboelectric energy harvesting. (a) Passive diode rectifier. (b) Diode-connected gated rectifier. (c)  Cross-coupled rectifier. (d) Active-diode rectifier. Energy harvesting. TENG, triboelectric generator.

A standard FBR configuration uses four *p*–*n* junction-based diodes, depicted in **Figure** 4a. For on-chip applications, a gated diode rectifier utilizing diode-connected transistors is suggested, as illustrated in Figure 4b. This approach represents the most straightforward method of integrating an FBR on a chip, although it encounters several limitations. Primarily, the power-conversion efficiency of this arrangement is limited in low-voltage systems due to the forward voltage drop across the diode-connected transistors.^19^ Additionally, the leakage in weak inversion of transistors can significantly impact low-power energy-harvesting systems. To reduce the conduction loss associated with transistors, an alternative approach previously popular in piezoelectric energy harvesting is applicable, which is the cross-coupled rectifier, or negative voltage converter,^20^ shown in Figure 4c. This design significantly reduces voltage drops, although it could experience issues with reflux current when the input voltage falls below the output voltage. Another design is the active-diode configuration, represented in Figure 4d, where one pair of NMOS transistors in the cross-coupled rectifier is substituted with transistors controlled by comparators. This adjustment allows the comparator to minimize the conduction loss on the transistors and improve current flow ability. Nonetheless, this type of rectifier requires a separate voltage supply to operate the amplifiers.^21^

### Active rectifiers

In contrast to passive rectifiers, active rectifiers utilize active switches that result in lower conversion losses. Although these converters require additional gate drivers and control circuits, they effectively address the polarity differences between voltage and current outputs of TENGs. This capability significantly enhances the power output, often multiplying the extracted power several times over that of a conventional passive energy-harvesting circuit.

**Figure 5:**
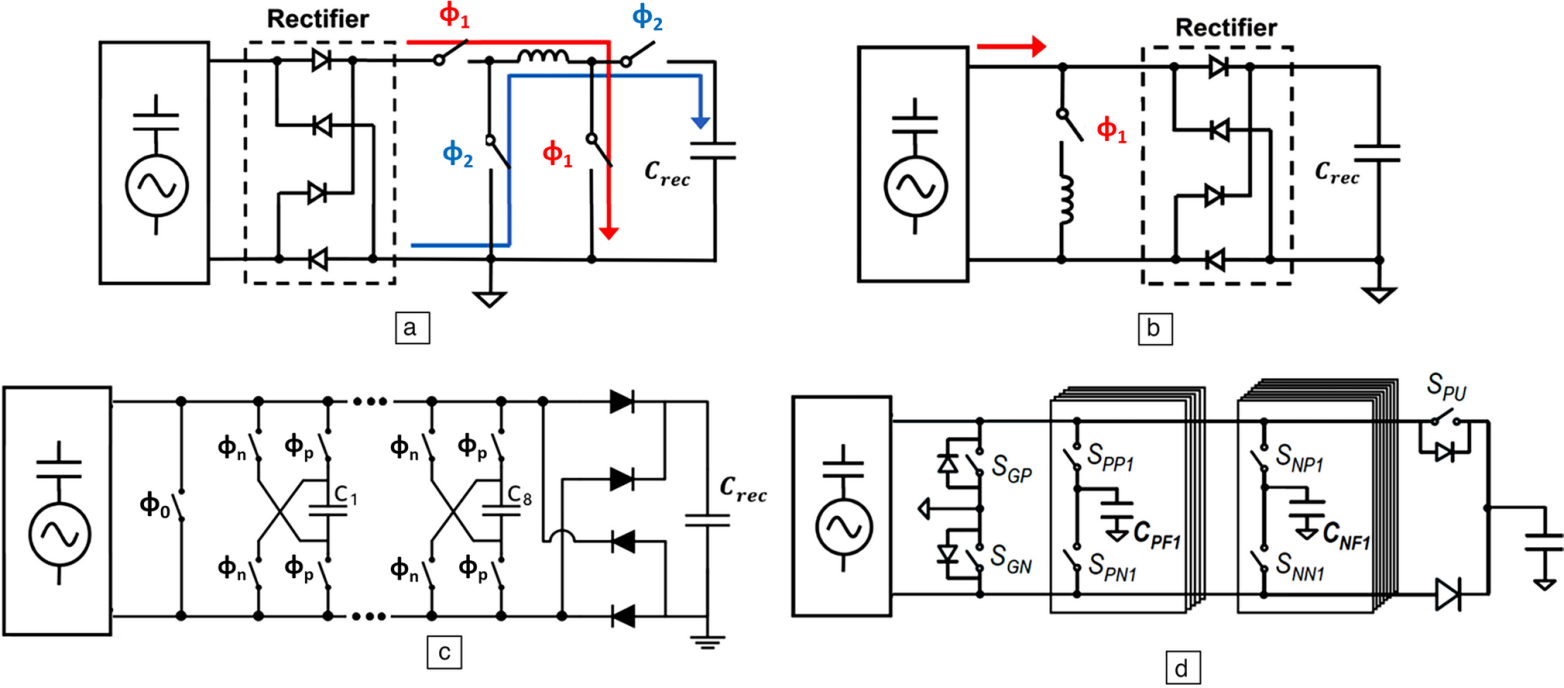
Widely used active rectifiers: (a)  synchronized electrical charge extraction; (b) synchronized switch harvesting on inductor; (c) synchronized switch harvesting on capacitors (SSHC); (d) dual-SSHC.

Among active rectifiers, the synchronized electrical charge extraction (SECE) rectifier is widely used to enhance energy extraction efficiency and was originally developed for use in piezoelectric systems.^22^ The standard circuit layout and voltage wave form of SECE are shown in **Figure** 5a. Differing from traditional energy-harvesting circuits, this rectifier connects directly to the voltage regulator without a capacitor connected to the output of the FBR. Most of the time, the transducer remains cut off from the FBR, resulting in an output voltage that mirrors the open-circuit voltage. At the voltage peak, energy is swiftly extracted via the phase Φ1. This process extracts all accumulated charges on the internal capacitor, resetting the voltage at the end of Φ1 and eliminating any polarity difference between the current and voltage outputs. In the following semi-cycle, the energy momentarily stored in the inductor is transferred to the capacitor *C*_*rec*_ along the phase Φ2.^23^ This method significantly boosts energy output by harvesting the charge at a higher voltage compared to standard circuits. The quadra-switch configuration in SECE functions as a buck-boost converter, allowing its output to potentially power electronic devices directly.

The synchronized switch harvesting on inductor (SSHI) technique, also known as bias-flipping, was developed to reduce energy losses that occur during the charging and discharging of a TENG's internal capacitance. The standard circuit for SSHI is depicted in Figure 5b. Here, when the I_P_ current direction changes, the switch is activated, causing the source voltage, previously equal to the rectification voltage, to be flipped by the inductor.^24^^,^^25^ The voltage-flipping phase, Φ1, ends when the inductor’s current reaches zero, at which point the switch is deactivated. The SSHI was first implemented in triboelectric energy harvesting in a discrete circuit in Reference 26. Subsequently, an on-chip SSHI rectifier for triboelectric energy harvesting was designed, featuring a combination of an inductive step-down converter and a switched-capacitor DC–DC converter arranged in series to manage voltage conditioning.^27^A double-stacked-chip SSHI that incorporates an intrinsic fractal switched-capacitor converter (FSCC) is detailed in Reference 28. This configuration improves energy harvesting by increasing the voltage tolerance of the harvester, achieved through the use of two series-connected full bridge rectifiers (FBRs) and storage capacitors positioned on two stacked chips.

Although the SSHI technique offers several benefits, it cannot be fully integrated on-chip due to the need for an external inductor. The inductor also introduces complications such as challenging control over the flipping time and fluctuating inductance, which can result in energy-conversion losses^29–31^ Consequently, an alternative approach without an inductor, known as the synchronized switch harvesting on capacitors (SSHC), was developed using the switched-capacitor technique^32–34^ as presented in Figure 5c. To attain higher efficiency, multiple capacitors are required to smoothly flip the voltage and minimize charge-sharing losses, achievable through additional parallel stages.

However, due to the varying inherent capacitance of the TENG (C_TENG_), the standard SSHC circuit is less efficient in triboelectric energy harvesting compared to piezoelectric applications, necessitating a redesign for optimal performance in triboelectric contexts. A dual-SSHC rectifier was designed to flip the TENG voltage with two individual capacitor banks to match the different C_TENG_ at different flipping moments,^35^ as shown in Figure 5d. In addition to the dual-SSHC rectifiers, an electrostatic charge boosting (ECB) rectifier was proposed to enhance the electrostatic energy extraction, making use of the varying C_TENG_.^36^ With the ECB technique, the output power from the rectifiers quadratically increases with the output voltage, enhancing energy extraction performance.

### Modeling and analysis for power optimization

A good power management system (PMS) for any kinetic energy harvester must maximize the amount of energy delivered to an electrical load in a minimum amount of time. For electrostatic transducers such as TENGs, the PMS must first dynamically bias the TENG with its optimum DC voltage, knowing that the converted power is proportional to the square of this voltage. This is the objective of the first stage of the conditioning circuit, which rectifies the TENG output signal into a DC voltage.

**Figure 6:**
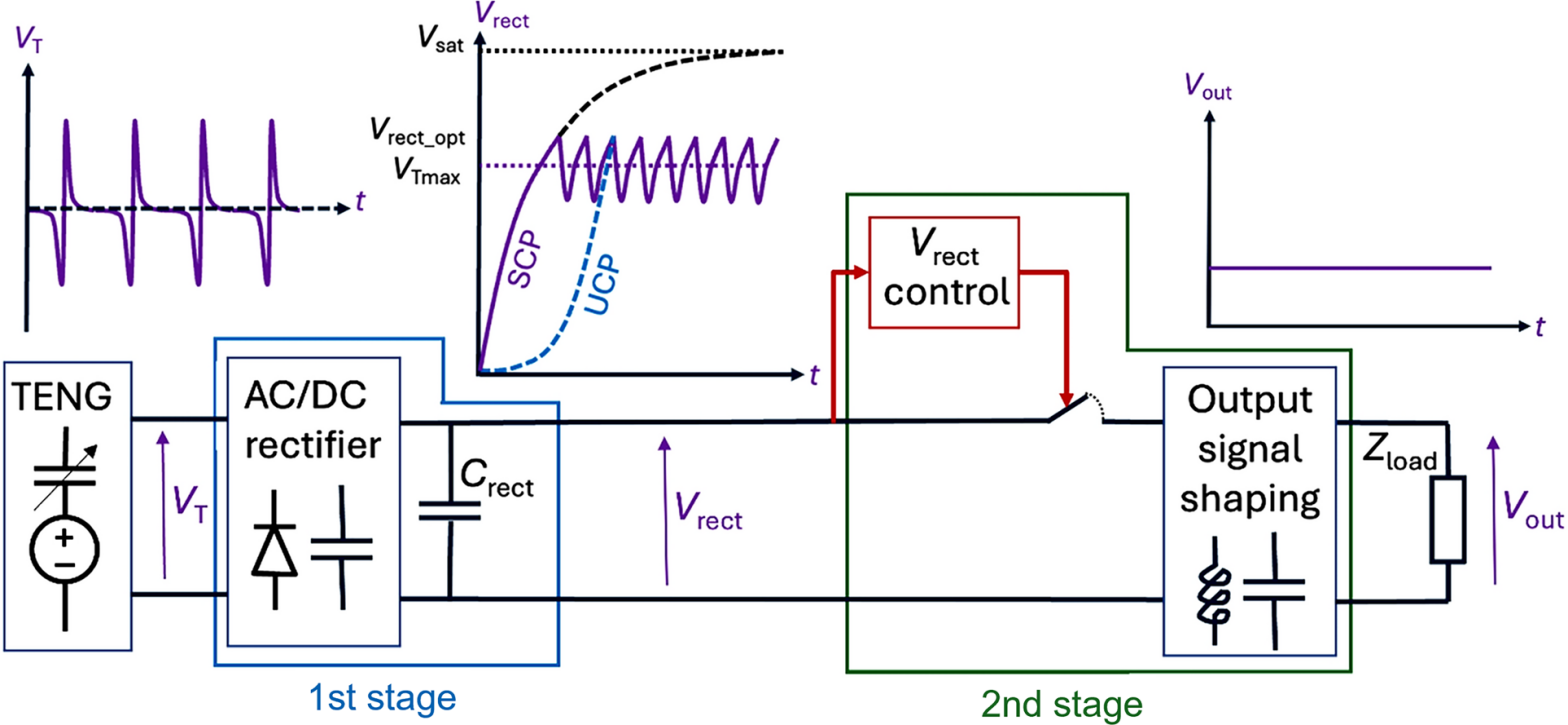
Ideal power management system for triboelectric nanogenerators (TENGs). The first stage reaches the optimum conversion point, and the second stage maintains the maximum power operation and generates the expected output signal. SCP, stable charge pump; UCP, unstable charge pump.

Depending on the situation, the harvested charges are transferred directly to an electrical load or to a second stage to reduce and stabilize the output voltage. Therefore, a PMS for TENGs typically has two stages: a first stage to maximize the harvested energy by maintaining a high DC voltage across the TENG, and a second stage to shape the output signal to an appropriate voltage for the targeted application, as illustrated in **Figure** 6.

### First stage conditioning circuit: Signal rectification

The first step in a typical TENG PMS is to generate a DC voltage from the TENG’s AC output signal. For most TENGs that can be modeled by a voltage source in series with a variable capacitor,^37^^,^^38^ this is achieved through the use of charge pump circuits that will progressively accumulate the electrical charges generated by the TENG with each mechanical cycle in a (relatively) large capacitor (referred to as ${C}_{rect}$ in the following). When the accumulated energy in ${C}_{rect}$ reaches the desired value, a switch is activated to extract the accumulated energy or a part of it.

The law of evolution of the voltage across ${C}_{rect}$ is usually given by ${V}_{rec{t}_{i+1}}=\upalpha {V}_{rec{t}_{i}}+\upbeta,$ where $\upalpha$ and $\upbeta$ are coefficients dependent on the parameters of the first stage, including the TENG and its rectifier circuit, and ${V}_{rec{t}_{i}}$ being the output voltage at the $i^{th}$ cycle. The α and $\upbeta$ values can be obtained from the study of the electrical network implementing the first stage.

### Stable charge pump rectifiers

As the number of mechanical actuation cycles *i* increases, if $\upalpha <1$ the voltage ${V}_{rect}$ across ${C}_{rect}$ gradually reaches a constant maximum voltage ${V}_{sat},$ which means that the charge pump is saturated. This is the case with charge pumps such as half-wave (HW) and full-wave (FW) diode bridge rectifiers. We usually call these rectifiers stable charge pumps (SCPs), and one of the objectives of the PMS is to avoid reaching saturation by activating the switch at the optimum moment. At saturation, the energy stored in ${C}_{rect}$ becomes constant with time, so the power generated is zero. In SCP α is close to 1, especially if ${C}_{rect}$ is large relative to the value of the TENG's minimum capacitance ${C}_{{T}_{\text{min}}}$, and $\upbeta$ is proportional to the TENG's internal DC bias voltage ${V}_{TE}$, which also corresponds to the average value of the surface voltage of the triboelectric layer.

Therefore, with an SCP, ${C}_{rect}$ must be discharged before the saturation occurs by activating a switch. The switch actuation mechanism can be of several types: electronic,^39^ electrostatic^15^ or mechanical.^40^ It is shown that the value of ${V}_{rect}$ that maximizes electromechanical conversion is given by ${V}_{rec{t}_{opt}}=\frac{\upalpha \upbeta }{1-{\upalpha }^{2}}$, which reduces to ${V}_{rec{t}_{opt}}=\frac{{V}_{sat}}{2}$ for common half- and full-wave diode bridges. This means that the PMS should turn on the switch just above $\frac{{V}_{sat}}{2}$ with these circuits, and turn it off just below, creating a narrow hysteresis around ${V}_{rec{t}_{opt}}$. However, if the PMS cannot control the switch actuation, the switch actuation follows a full hysteresis so that ${C}_{rect}$ is fully discharged each time the switch is actuated. The optimum switch-on voltage then becomes ${V}_{rec{t}_{opt}}=0.7\,{V}_{sat}$, but the energy harvested would be significantly less than that of a switch with narrow hysteresis with optimum actuation.

Thus, the optimum voltage for discharging ${C}_{rect}$ is between 0.5 and 0.7 ${V}_{sat}$, depending on the hysteresis level of the switch. However, all this assumes an ideal lossless switch. With a narrow hysteresis, the switch is activated much more often than with a full hysteresis. If it has significant losses, the narrow hysteresis switch may not be the most efficient choice.

During the first few conversion cycles, when ${V}_{rect}$ is much lower than ${V}_{TE},{V}_{rect}$ increases twice as fast with the FW rectifier than with the HW. Consequently, if the energy harvested must be used quickly at a low value of ${V}_{rect}$, the FW rectifier should be chosen. Conversely, if the PMS manages to maintain ${V}_{rect}$ at a higher value, the HW rectifier should perform better: in terms of harvested energy, the HW rectifier will outperform the FW by a factor of $\upeta +1$ at the optimum value $\frac{{V}_{sat}}{2}$, where $\upeta =\frac{{C}_{{T}_{\text{max}}}}{{C}_{{T}_{\text{min}}}}$ is the ratio of maximum to minimum capacitance of the TENG.

Alternatively to passive SCP, circuits based on active charge extraction such as synchronous electrical charge extraction (SECE) or synchronous switching harvesting on inductor (SSHI),^22^ can be implemented. They can directly control the level of the output DC voltage without the need of a second stage in the PMS. However, they also require a switch that needs to be accurately actuated several times per mechanical actuation, which makes them complex and power consuming,^41^ unless a dedicated design is implemented in a CMOS-integrated technology like in Reference 27.

### Unstable charge pump rectifiers

If $\upalpha >1$, ${V}_{rect}$ increases exponentially with the time without saturation, and so does the energy harvested and the power available. We call these circuits unstable charge pump (UCP) rectifiers. Of course, the exponential increase in ${V}_{rect}$ will only last until losses occur in the PMS due to the excessively high voltage (e.g., increased reverse current in the diodes), or due to unwanted electrostatic discharges occurring in the TENG. This exponential increase of ${V}_{rect}$ will occur on condition that a constraint on $\upeta$ is met, a constraint that will depend on the UCP architecture chosen. UCPs can, therefore, achieve a high level of energy conversion, even with poor quality triboelectric materials, but the number of mechanical cycles required to outperform an SCP rectifier can be very long.^42^

The choice of the best UCP architecture is strongly determined by the value of $\upeta$. If $\upeta$ is less than 2, it is preferable to implement the low capacitance variation (LCV) architecture proposed by Lefeuvre et al.^43^ For $\upeta$ greater than 2, it is generally preferable to use the cascade Bennet's doubler (CBD) proposed by Karami et al.^44^ The optimum number of multiplier cells for each architecture will depend on the values of $\upeta$, ${V}_{TE}$, and the maximum voltage supported by the system.^45^

### Second stage conditioning circuit: Step-down DC-to-DC voltage

With the UCP rectifier, the higher the value of ${V}_{rect}$, the greater the energy conversion. In addition, for all SCP rectifiers, as we mentioned earlier, the energy harvested at the optimum cycle is proportional to ${{V}_{TE}}^{2}$. This is why optimizing the triboelectric material to maximize the charge trapped during triboelectric contact, and then maximizing its surface voltage ${V}_{TE}$, has been one of the key objectives in achieving an efficient energy collector. As a result, ${V}_{TE}$ is now routinely greater than 100 V for most TENGs. Knowing that for the FW rectifier the optimum value of ${V}_{rect}$ is proportional to ${V}_{TE}$ and is proportional to $\upeta {V}_{TE}$ for the HW,^46^ the target value of ${V}_{rect}$ can be very high, well above the DC voltage needed to power most applications that are typically around a few volts.

In this case, a second stage, acting as a DC–DC voltage converter, must be used. The first stage accumulates the charges collected in ${C}_{rect}$, while the second stage transfers part of these charges to a larger capacitor through an inductor, activating a floating switch at a frequency much lower than the mechanical frequency.^47^ This circuit is similar to a buck DC–DC converter, except that the switch is only activated when the maximum voltage allowed by the PMS is reached or, in the case of SCPs, around ${V}_{rec{t}_{opt}}$. The second stage of the PMS, therefore, has two roles: (1) to optimize power-conversion efficiency by maintaining a high/optimal DC bias voltage across the TENG, and (2) to adjust the PMS output voltage ${V}_{store}$ to the value required by the system to be powered.

If the expected actuation voltage is less than ~300 V, the switch must be electronically controlled, preferably with a dedicated CMOS IC, to minimize interference and additional power consumption. For higher voltages, a plasma switch consisting of two conductors separated by a few microns can be used. When ${V}_{rect}$ exceeds the limit defined by Paschen’s Law, an electrostatic discharge occurs between the two electrodes of the switch, transferring charges from ${V}_{rect}$ to ${V}_{store}$. If one of the switch electrodes is mobile, using MEMS technology, for example, very narrow hysteresis can even be achieved without the need for controlled electronics.^48^

## Conclusion

In recent years, there have been significant advancements in TENG technology across the dimensions of device, circuit, and system design, each contributing to enhanced capabilities in energy harvesting. At the device level, advanced mechanical and structural designs have substantially boosted the electrical output and operational efficiency of TENGs, pointing toward a future where these devices could see broader application in diverse environments. In circuit design, the adoption of configurations such as SSHI, SSHC, SECE, and other passive/active rectification circuits has markedly improved energy-conversion efficiency. Further research on integrating maximum power point tracking (MPPT) into rectification circuits is essential to elevate output power to new levels. At the system level, effective voltage biasing and impedance matching ensure that TENG devices and rectification circuits operate in harmony, optimizing overall system performance. Effective power management is fundamental to TENG applications, as it enables consistent, reliable energy capture and regulation, paving the way for scalable integration across various real-world scenarios.
